# Abnormal amplitude of low-frequency fluctuations associated with rapid-eye movement in chronic primary insomnia patients

**DOI:** 10.18632/oncotarget.17921

**Published:** 2017-05-17

**Authors:** Qian Ran, Jia Chen, Chuan Li, Li Wen, Faguo Yue, Tongsheng Shu, Jianxun Mi, Guangxian Wang, Lei Zhang, Dong Gao, Dong Zhang

**Affiliations:** ^1^ Department of Radiology, The Second Affiliated Hospital of The Third Military Medical University, Sha Pingba, Chongqing 400037, China; ^2^ Department of Sleep and Psychology, Institute of Surgery Research, The Third Affiliated Hospital of The Third Military Medical University, Da Ping, Chongqing 400042, China; ^3^ College of Computer Science and Technology, Chongqing University of Posts and Telecommunications, Chongqing 400065, China

**Keywords:** insomnia, REM, ALFF, resting-state fMRI, PSG

## Abstract

**Purpose:**

Chronic primary insomnia (CPI) is the most prevalent sleep disorder worldwide. CPI manifests as difficulties in sleep onset, maintaining sleep, prolonged sleep latency, and daytime impairment and is often accompanied by cognitive problems such as poor academic performance, poor attention, and decreased memory. The most popular explanation of insomnia is hyperarousal or increased activities of neurons. Rapid eye movement (REM) sleep detected by polysomnography (PSG) exhibits a positive relationship with brain homeostasis and can be helpful for optimally preparing an organism for emotional and social function. Limited work has been performed to explore brain function of insomnia patients in combination with PSG analysis.

**Results:**

We observed increased ALFF within areas related to hyperarousal such as the midbrain and bilateral extra-nucleus, whereas decreased ALFF was observed within areas associated with memory and attention involving the parietal and occipital lobule and others. Furthermore, the altered ALFF was associated with the duration of insomnia, sleep efficiency, duration of REM, latency of RME and ratio of REM.

**Materials and Methods:**

In this study, we recruited twenty-five CPI patients and twenty-five normal sleep (NS) volunteers as a control group to investigate the amplitude of low-frequency fluctuations (ALFF) and the correlation between those altered ALFF regions through resting-state fMRI and PSG data.

**Conclusions:**

These findings suggest that hyperarousal reflected by ALFF abnormality within brain areas related to cognition and emotion in insomnia associated with REM sleep.

## INTRODUCTION

Chronic primary insomnia (CPI) is the most prevalent sleep disorder. CPI manifests as difficulties in sleep onset, falling asleep, maintaining sleep, prolonged sleep latency and daytime impairment [1]. Approximately 10–15% of the population worldwide suffers from CPI [2, 3]. In China, 45% of the population suffers from occasional insomnia because of work pressures, urbanization and industrialization, as surveyed by the Chinese Medical Association (CMA) [4]. Abundant sleep can facilitate the removal of free radicals from the brain produced during wakefulness [5]. However, when sleep loss, interruption or disturbance occurs, the accumulated free radicals cannot be removed, and the biological characteristics of long-distance signal transmission and nerve cell function cannot be recovered in a timely manner [6, 7]. Chronic insomnia is accompanied by cognitive and conduct problems, such as poor academic performance, poor attention, and decreased memory [8–11]. Moreover, CPI patients usually also accompanied with emotional related problems, such as depression, anxiety, hysteria and so on [12–15]. Most depression patients were accompanied with insomnia, and patients with depression or anxiety frequently visit outpatient departments solely due to insomnia [16]. Hence, CPI patients may appear emotional related problems such as depression in later stages in the absence of early-stage diagnosis and efficient treatment.

Polysomnography (PSG) is an objective method that is normally used to monitor sleep non-rapid eye movement (NREM) and rapid eye movement (REM) in the clinic. REM sleep homeostasis has been reported to have a positive relationship with brain homeostasis and can be helpful for optimally preparing an organism for emotional and social function [17]. Some studies reported insomnia patients REM stage sleep are abnormal [18].Therefore, we assumed abnormal REM sleep may be correlated with insomnia. Previous neuroimaging studies have mainly focused on regional brain metabolism or brain activity using single-photon emission computed tomography (SPECT), positron emission tomography (PET) and magnetic resonance imaging (MRI) methods [19–21]. However, limited work has been performed to explore the relationship between functional MRI in CPI and objective sleep indices measured by PSG.

The most popular explanation of insomnia is hyperarousal or increased activities of neurons, metabolism in special areas of the brain and excretion by neuroendocrine systems [22]. Some researchers have proposed that subjects with CPI show a state of arousal and frequently experience intrusive worrisome or negative thoughts [23]. Therefore, people suffering from CPI find it difficult to initiate sleep or return to sleep after awakening due to this intrusive hyperarousal state [12]. In a previous SPECT study, CPI patients showed hypoperfusion in preselected regions, including the basal ganglia (the most decreased area), medial frontal cortex, occipital and parietal cortex, compared to good sleepers during sleep [19]. Nofzinger [20] used the PET method and found that patients with insomnia displayed increased global metabolism of glucose during both sleep and wakefulness. Moreover, patients with insomnia showed a smaller decrease in glucose cost from wakefulness to sleep in ascending reticular activating systems, the thalamus, hypothalamus and some regions related to cognition and emotion such as the hippocampus, insular, amygdala, prefrontal cortex and the anterior cingulate cortex [20]. Despite these findings, a consensus on the brain activity alterations of insomnia patients is lacking, but it is likely that insomnia is associated with regional metabolic abnormalities in regions of the brains and that these abnormalities are closely related to emotion and arousal.

Resting state functional magnetic resonance imaging (rs-fMRI) is an effective, non-radiation and non-intrusive tool that is broadly used in neurology research [24]. rs-fMRI can detect spontaneous neuronal activity and provide new insights on the pathophysiology of diseases [25]. Many neurological or mental diseases exhibit dysfunction of several distinct areas of the brain simultaneously. Previous studies have revealed synchronous activity of neurons as a necessary condition to exactly integrate and coordinate information processing [26]. Deterioration of information processing abilities and abnormalities in brain functions are observed if changes in neuronal or distinct brain synchrony occur [27]. The amplitude of low-frequency fluctuations (ALFF) is an index and its value is the average square root of power of spectrum, which is used to detect the regional spontaneous neuronal activity by rs-fMRI [28, 29]. ALFF reflects physiological signals and was higher in grey matter [24], It’s simple, easily implemented, reliable, and robust and exhibits good-to-moderate test-retest [30, 31], Fransson and Horovitz et al. applied ALFF to detect functional modulations and pathophysiological changes while awake and during light sleep [32, 33]. ALFF has been observed in brain areas associated with cognition and emotion, such as the dorsolateral prefrontal cortex, occipital gyrus and limbic system, in insomnia patients [34]. Hence, ALFF detected by rs-fMRI is helpful for exploring alterations of brain activity in neurological or psychosomatic diseases. However, the correlations between ALFF alterations and REM sleep have not been reported.

Thus, the aim of this study was to investigate alterations of ALFF in CPI patients and explore how these changes correlate with REM sleep. Based on previous findings, we hypothesized that CPI patients will show abnormal ALFF in brain areas related with wakefulness, emotion or cognition and that these abnormalities would be associated with REM sleep indices measured by PSG.

## RESULTS

Only 21 insomnia patients and 20 normal sleep participants were included in the analysis due to those nine participants head movement were exceeded head movement criterion (any part of volume in those nine participants: any of X, Y and Z direction translation was more than 2 mm; and any of roll, pitch and raw direction rotation was more than 2 degrees). Also there were no significant different head movements (including mean translation and mean rotation) between two groups.

### Descriptive behavioural data

The characteristics of the participants in the CPI group and NS group are listed in Table 1. Two-sample *t*-test showed no significant differences in age and SAS scores between the two groups: *t*_(39)_ = −0.85, *p* = 0.4; *t*_(39)_ = −1.73, *p* = 0.09; Chi-Square Test showed there was no significant difference in gender: χ^2^*=0.2, p* = 0.66. However, the groups differed in education and PSQI scores: *t*_(39)_ = 2.48, *p* = 0.02, t; *t*_(39)_ = 15.07, *p* < 0.001.

**Table 1:** Descriptive behavioural data

	NS Group (20)		CPI Group (21)		Two-sample *T* Test	
	Mean	SD	Mean	SD	*t* value	*p*
Age (years)	38.65	7.40	40.62	7.52	−0.85	0.40
Education (years)	15.55	3.61	12.76	3.60	2.48	0.02
SAS	37.40	8.12	41.33	6.32	−1.73	0.09
PSQI	2.85	0.99	13.33	3.02	15.07	< 0.001

	NS Group (20)		CPI Group (21)		Chi-Square Test	
Gender(F/M)	F	M	F	M	Pearson Chi-Square	P
	14(70%)	6(30%)	16(76%)	5(24%)	0.2	0.655

F indicates female; M indicates male.

### PSG data

The examination of the sleep state of the CPI participants is presented in Table 2. All CPI patients exhibited sleep structure abnormality, and some also exhibited difficulty in falling asleep.

**Table 2:** Polysomnographic (PSG) monitoring results for the CPI group

	Time of Insomnia (years)	Percent sleep efficiency (% SE)	Latency of sleep (min)	Latency of REM (min)	Duration of REM (min)	Ratio of REM (%)
Sub1	6	54.1	18	275	10.5	3.2
Sub2	20	76.6	34	95	55.5	13.5
Sub3	1.5	62.8	43	92.5	17	4.8
Sub4	3	69.4	52.5	100	34.5	8.8
Sub5	3	73.2	60	75	64.5	16.1
Sub6	1	77.8	42.5	90	67.5	15
Sub7	10	65.1	2.5	91	36	9.9
Sub8	3	86.9	13.5	110.5	57	12.8
Sub9	2	77.5	1.5	105.5	81	18.1
Sub10	10	57.8	16	125	58	18.4
Sub11	6	87	11	29.5	168.5	32.4
Sub12	10	68.8	33	201	75.5	22.4
Sub13	7	-	37.2	21.2	114.5	23.3
Sub14	3	72.2	26.5	59	105.5	26.8
Sub15	3	58.4	10.5	141	53	16.2
Sub16	7	65.8	10	65.6	75.5	23.6
Sub17	6	89.4	19	85	80.5	17.5
Sub18	6	81.9	29	262	70.5	16.1
Sub19	5	-	66	60	68	16.4
Sub20	14	87.9	6.5	60.5	73	16.4
Sub21	2	69.3	8.5	67.5	21.5	5.8

REM, Rapid-eye movement.

### ALFF alterations in the CPI group compared with the NS group

**Figure 1:**
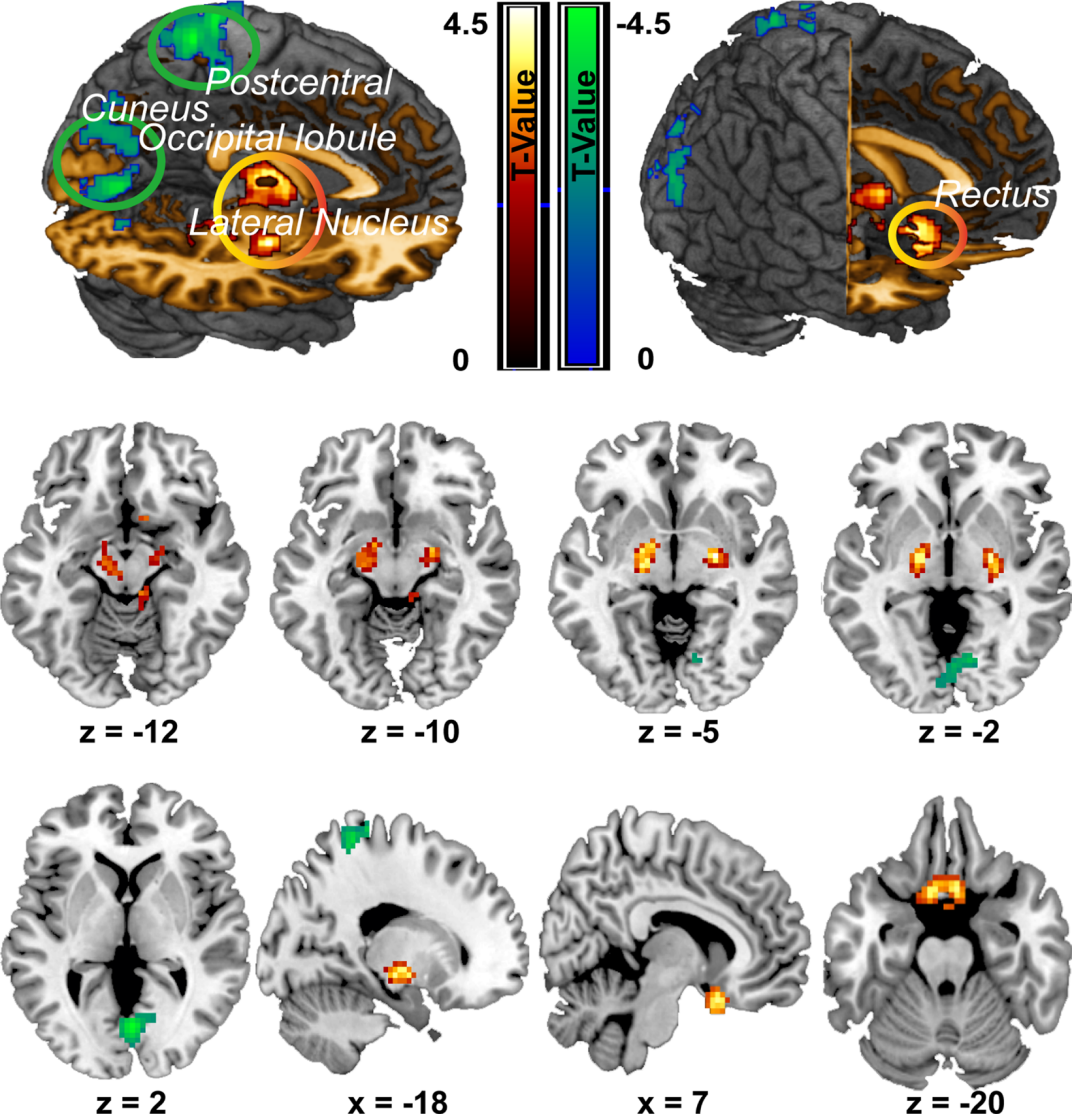
ALFF differences between the CPI group and NS group Decreases in ALFF in the CPI group are highlighted in green, and increases in ALFF in the CPI group are highlighted in yellow. However, midbrain (z = −12, z = −10) was not survived after correction when GM added as a covariate.

Compared with the NS group, the CPI group showed significantly increased ALFF in two clusters. The first cluster primarily included regions of the bilateral rectal gyrus (peak MNI coordinate: -9, 12, -24; *t* = 4.37, cluster size =224), while the second cluster included the bilateral lateral-nucleus and midbrain (peak MIN coordinate: 24, -9, -6, t = 5.27, cluster size = 213 voxels). However, the midbrain in the second cluster did not show significant difference any more after GM added as a covariate for two groups comparison analysis. Conversely, the CPI group showed significantly decreased ALFF compared with the NS group mainly in three clusters. The first cluster mainly included the left superior parietal lobule and left postcentral gyrus (peak MNI coordinate: -21, -42, 66; *t* = 4.60; cluster size = 218); the second cluster was mainly located in the bilateral cuneus (peak MNI coordinate: 3, -72, 3; *t* = 4.52, cluster size = 406); and the third cluster mainly included the left middle and inferior occipital lobule (peak MNI coordinate: -27, -87, 15; *t* = 3.7, cluster size = 206) (Figure 1; Table 3).

**Table 3:** ALFF alterations in the CPI group compared with the NS group

	ALL Brain Regions	Peak MNI Coordinates			Cluster (number of voxels)	Peak *T*-value
		x	y	x		
ALFF Decrease	Postcentral gyrus_L/ Parietal_Sup_L/ Paracentral_Lobule_L	−21	−42	66	218	4.60
	Cuneus_L/R	3	−72	3	406	4.52
	Occipital_Mid_L/ Occipital _Inf_L	−27	−87	15	206	3.70
ALFF Increase	Rectus _L/R	−9	12	−24	224	4.37
	Lateral_nucleus_L/R Midbrain	24	−9	−6	213	5.27

However, midbrain was not survived after correction when GM added as a covariate.

### Correlations of regional ALFF alterations with PSG

**Figure 2:**
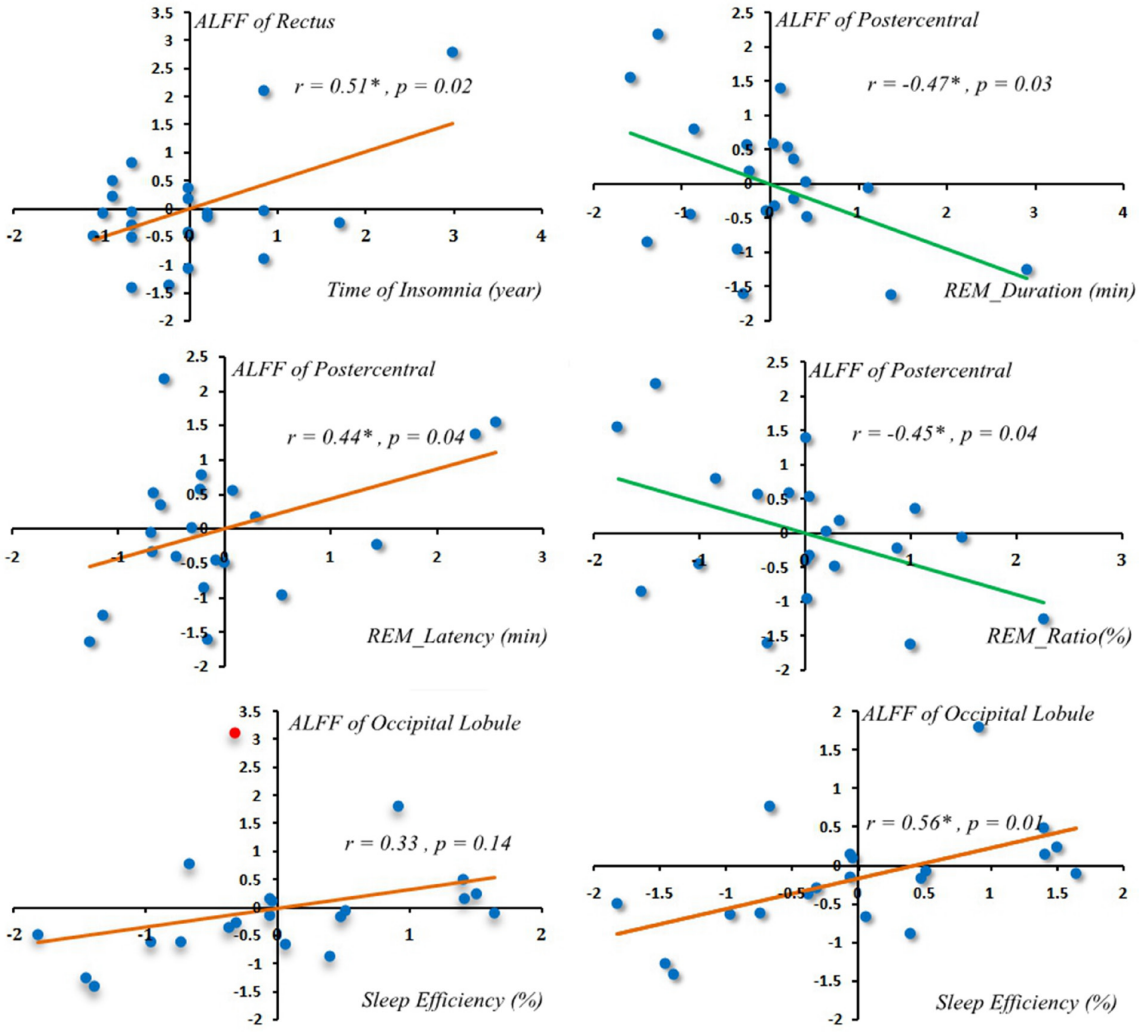
Correlations of regional ALFF alterations with PSG One patient occipital lobule ALFF Z value more than 3 (the red spot indicated ALFF Z value more than 3 in the left scatterplot at the bottom row). Occipital lobule showed positive correlation with sleep efficiency after that abnormal ALFF Z value removed (Pearson correlation = 0.56, *p* = 0.01) (in the right scatterplot at the bottom row).

Only between rectus gyrus, postcentral gyrus and time of insomnia, latency of REM, duration of REM, ratio of REM showed correlated. ALFF alterations in the rectus gyrus was positively correlated with time of insomnia (Pearson correlation coefficient = 0.51, *p* = 0.02) (Figure 2). ALFF alterations in the postcentral gyrus were positively correlated with latency of REM sleep (Pearson correlation coefficient = 0.44, *p* = 0.04) (Figure 2) but were negatively correlated with the duration of REM sleep (Pearson correlation coefficient = −0.47, *p* = 0.03) (Figure 2) and the ratio of REM sleep (Pearson correlation coefficient = −0.45, *p* = 0.04) (Figure 2). One patient ALFF *Z* value in occipital lobule showed greater than 3. Occipital lobule showed positive correlation with sleep efficiency which not shown correlation before this abnormal ALFF *Z* value removed (Pearson correlation coefficient = 0.56, *p* = 0.01) (Figure 2).

## DISCUSSION

The goal of the current study was to explore the alterations of ALFF in CPI patients and investigate whether these alterative ALFF regions had any correlation with PSG index and insomnia related index. The main findings are as follows. First, CPI patients showed not only increased ALFF in the bilateral lateral-nucleus, midbrain and bilateral rectal gyrus but also decreased ALFF in the left superior parietal lobule, left postcentral gyrus and left occipital area (including the left middle and inferior occipital lobule and bilateral cuneus). Second, the changes in the ALFF in the rectal gyrus were positively correlated with the time of insomnia, the changes in the ALFF in the postcentral gyrus were positively correlated with the latency of REM sleep and negatively correlated with the duration of REM sleep, and the changes in ALFF in the postcentral gyrus were negatively correlated with the ratio of REM sleep. The changes in ALFF in occipital lobule showed positive correlation with sleep efficiency after one abnormal ALFF Z value was removed. However, these regional ALFF changes were not correlated with PSQI scores. These results offer direct neuroimaging evidence for ALFF changes associated with insomnia and, in particular, changes correlated with REM sleep. In this part, we discussed the most meaningful regions associated with insomnia.

Consistent with our hypothesis, abnormalities of ALFF related to wakefulness regions were observed in insomnia patients. Many previous studies and long-standing observations have demonstrated that “hyperarousal” is the main cause of insomnia [35]. The main arousal system or activating system is the ascending reticular activating system (ARAS), which is located in the brainstem (medulla oblongata, pons and midbrain) and plays an important role in arousal and sleep. ARAS dysfunction results in abnormal sleep, including insomnia, coma or hypersomnia [36, 37]. ARAS not only modulates sleep-wake states but also enables appropriate reactions to the surrounding environment [38]. ARAS is excited by stimulation from the surrounding environment, and the stimulation signal projects from the ARAS to the cerebral cortex. Feedback or information on how to react to the stimulus is integrated by the cortex and sent to the spinal cord via the descending reticular activating system (DRAS), finally resulting in changes in body posture induced by spinal cord instruction and an adapted reaction to the stimulus. Any disturbance of ARAS activity would damage this process. Nofzinger [20] discovered that wake-promoting ARAS activity and thalamus glucose metabolism were increased in primary insomnia patients. ARAS hypermetabolism would increase or heighten emotional activities and produce an arousal state and insomnia. In our study, we detected increased midbrain ALFF in the insomnia patient group compared with the normal sleep group. The midbrain, as part of the brainstem, is a component of the ARAS. The increase in ALFF in this region indicates increased ARAS activity. The projection from the ARAS to the cerebral cortex occurs through the thalamus. Here, we also observed increased ALFF in the lateral-nucleus, a part of the thalamus consisting of the lateral dorsal nucleus and lateral posterior nucleus that is considered an integration nucleus, in insomnia patients. Our findings support a hyperarousal state as the core cause or core predisposing factor for insomnia. Moreover, ALFF increased not only in brainstem ARAS but also in the lateral-nucleus, a vital part of the pathway between the ARAS and the cortex. After GM volume added as a covariate the midbrain ALFF showed no significance between two groups at correction level. It implied midbrain activity was sensitive to cortex structure. Previous study demonstrated long-time poor sleep was correlated brain cortex atrophy and increased this rate [39]. Thus, dysfunction of the ARAS and related pathway components may be the underlying mechanism of insomnia.

Interestingly, we observed an increase in bilateral rectus gyrus ALFF in insomnia. The rectus gyrus is part of the ventromedial prefrontal cortex (VMPFC) which played a crucial role in the generation of arousal and insomnia and its functional activation altered was caused by sleep deprivation [34, 40, 41]. Prominent bilateral orbitofrontal cortex which also as a sub-region of prefrontal cortex [41] and rectus gyrus grey matter are smaller in elderly depression [42]. These observations are consistent with a close relationship between insomnia and mental diseases such as depression and suggest ALFF of the rectus gyrus should altered in insomnia. However, we observed increased ALFF in the rectus gyrus. We provide two explanations for this finding. First, this study excluded participants with moderate or severe class anxiety in the previous 1 month; such anxiety may influence brain function or structure, and thus the degree of anxiety or emotion was not considered as a factor in this research. Second, VMPFC is a region includes several sub-regions and is a area. Although its function showed abnormal in sleep deprivation, it did not described which sub-region function altered [40]. Still, rectus gyrus grey matter was reduced in elderly depression containing sleep problems, it may explained that the increased ALFF in the rectus gyrus may compensate for the long-term deficit of function of the prefrontal cortex and daytime fatigue. But how rectus gyrus morphology, function, and local activity affect each other needs more work to reveal this in next study.

In the current study, we also observed decreases in ALFF in the left superior parietal lobule, left postcentral gyrus, bilateral cuneus in the chronic insomnia patient group. The vital function of sufficient sleep for the brain is based on the appropriate regulation of brain function and brain development [1, 43, 44]. Chronic primary insomnia associated with working memory damage [45, 46] and spatial work memory deterioration [4]. The prefrontal lobe mediates working memory and stores information on spatial position [47, 48]. The superior parietal lobule projects and receives fibers from the prefrontal cortex, and the received fibers encode dynamic visual information about space and position and integrate these complex messages to form spatial working memory [49]. The prefrontal cortex and parietal cortex are activated during spatial memory tasks [47, 48], which mainly depend on the interaction between the parietal and frontal areas [50]. Any structural damage or abnormal activity in the prefrontal cortex or superior parietal lobule would disrupt this interaction. The prefrontal and parietal cortex were found regional hypometabolism after sleep deprivation [51]. The orbitalfrontal cortex is a prefrontal cortex in the frontal lobe. The bilateral orbitalfrontal cortex and the inferior parietal lobule showed ALFF decreased in short-term sleep deprivation people and parietal lobule [29, 52]. Li Yongli et al. detected decreased connectivity between the superior frontal gyrus and superior parietal lobule in insomnia patients [4]. In addition, orbitofrontal and parietal cortex grey matter volume are smaller [53] and the dorsolateral prefrontal cortex grey matter concentration is decreased in insomnia patients [54]. In addition to decreased functional connectivity decreased between the prefrontal cortex and the parietal lobule, the grey matter volume or grey matter concentration of these regions decreased. In support of the results of these previous studies, we observed decreased ALFF in the superior parietal lobule in this study. This complementary evidence indicates similar trends of alterations of structure, functional connectivity and regional activity in these regions that executed the same tasks or functions appear in insomnia.

Decreased ALFF was also observed in the left occipital lobe. Gamma-aminobutyric acid (GABA) is a vital inhibitory neurotransmitter and plays a significant role in mediation of sleep based on the sleep-wake regulation hypothesis [55, 56]. David T. Plante et al. detected an approximately 33% reduction of GABA production in the occipital cortex of unmedicated primary insomnia patients. This observation suggests that the hyperarousal state in insomnia is correlated with decreased GABA and that an imbalance between inhibitory and excitatory function appears if there is an abnormality of the excretion of any inhibitory or excitatory neurotransmission [20, 22, 23, 57]. We observed a decrease in left occipital lobule ALFF in insomnia. Also we found occipital lobule ALFF was positive correlated with sleep efficiency after one subject abnormal ALFF value was removed. Longer period poor sleep efficiency was associated with lower occipital lobule ALFF. We suppose that occipital lobule inhibition function was impaired in initiation of sleep due to the decrease in GABA in insomnia and poor sleep efficiency. These findings provide new insights for the exploration of the correlation between alterations of ALFF and GABA in future studies to achieve a better understanding of insomnia.

In this study, ALFF alterations in the rectus gyrus were significantly positively correlated with the duration or time of insomnia. Alterations in the postcentral gyrus were positively correlated with latency of REM sleep and negatively correlated with duration of REM sleep and ratio of REM sleep. Stable REM sleep provides support for brain function homeostasis, optimally prepares the organism for emotional and social functioning and regulated cognition in the coming day, and also benefits memory consolidation [17]. These alterations of ALFF and alterations associated with REM sleep reveals a richer underlying mechanism of insomnia.

In addition to the discovery of meaningful results, the limitations of the current study should not be ignored. First, a recent research study reported that gender may cause differences in regional ALFF [34]. The sample size in this study is small. In normal sleep: female was 14 (70%) and male was 6 (30%). In insomnia group: female was 16 (76%) and male was 5 (24%). Especially the rate of male was too small. Hence in this study we did not separately analyze. Of course, we chosen only female to compare (because about 70% was female) in this part. We found occipital lobule, cuneus and postcentral gyrus ALFF decreased in patient group. And rectus and midbrain showed increased in patient group. However only occipital lobule, cuneus and postcentral gyrus can survive after correction. The results were not significant after correction may caused by small sample size. Second, the time of insomnia is different in different patients. We did not do sub-group (different duration of insomnia) analysis in this study for small sample size as well. In the future study we will increase sample size to improve these analysis. Third, absence PSG in normal volunteers was indeed a weakness. All volunteers were asked to finish PSQI questionnaire and revised diagnostic criteria for sleep. All of them were belonged to this criteria. In future study, all normal volunteers will have PSG examination.

In conclusion, this study investigated alterations of ALFF in chronic insomnia patients. The results showed that ALFF increased in the bilateral nucleus, midbrain and rectus and decreased in the superior parietal lobule, postcentral gyrus, paracentral lobule, occipital lobule, cuneus and lingual lobule. Moreover, the alterations in the postcentral gyrus, rectus and occipital lobule were mainly associated with REM sleep index. These alterations may influence each other. Insomnia maybe is a brain network abnormality or circuit loop abnormality disease.

## MATERIALS AND METHODS

### Participants

#### Chronic primary insomnia (CPI) patients

A total of 25 primary insomnia patients were recruited from the department of Sleep and Psychology of the third affiliated hospital of The Third Military Medical University, Chongqing, China. The inclusion criteria were as follows: (1) adult patients diagnosed with CPI according to the Diagnostic and Statistical Manual of Mental Disorders, 4th Edition and PSG results; (2) absence of moderate or severe class anxiety in the last month; (3) no role of substance or medication abuse in insomnia; (4) no other sleep-related diseases, such as hypersomnia or parasomnia; (5) no additional mental disorders, central nervous system diseases, head trauma or psychiatric disorders; (6) neither pregnant nor breast-feeding. The patients’ clinical details are listed in Table 1.

#### Normal sleep participants (NS)

A control group of 25 normal sleep volunteers was recruited through advertisement from the second affiliated hospital of The Third Military Medical University. All volunteers were recruited under the revised diagnostic criteria for defining normal sleep controls [58]. In accordance with the 1964 Helsinki declaration and its later amendments, written informed consent for this study was obtained from all individual participants, and the study was approved by the Institutional Human Participants Review Board of the second affiliated hospital of The Third Military Medical University prior to the study. All participants were thanked for their time and received financial compensation after completing all examinations.

### Measures

### Behavioural assessments

To consider the effect of external emotion on sleep, all participants completed the Self-Rating Anxiety Scale (SAS) questionnaire, which was considered a reliable, ecologically valid and sensitive measurement for anxiety examination [59–61]. It contains 5 affective and 15 somatic symptoms related to anxiety [61]. Each item has a 4-point scale ranging from ‘none of the time’ to ‘most of the time’.

**Sleep assessments**

The CPI and NS groups all took the Pittsburgh Sleep Quality Index (PSQI) questionnaire [62]. Additionally, the NS group were subjected to the revised diagnostic criteria for defining normal sleep [58]. The PSQI contains 9 self-report items. Higher scores are indicative of poorer quality of sleep. Following the standard of Buysse et al. (1989), a PSQI score greater than or equal to ≥ 5 was considered poor sleep. This threshold has been demonstrated to have high sensitivity and specificity for differentiating insomnia and normal sleep [62]. Therefore, the PSQI score of all participants in the CPI group in this study should be greater than or equal to ≥ 5 and the NS group PSQI should be less than 5. The NS group was also evaluated using the revised diagnostic criteria suggested by Louise et al. [58]. This test mainly evaluates 4 items: sleep disruption, sleep disorders, circadian disruption and general health. All participants in the NS group had no sleep problems.

### Polysomnographic (PSG) monitoring

Only the CPI group underwent one-night PSG because most of the normal volunteers were not willing to stay overnight in the hospital to undergo the PSG examination. In the CPI group, the patient’s bed time and arising time were kept as close as possible to the patient’s usual sleep schedule at home. PSG was recorded following the American Academy of Sleep Medicine criteria [63]. The measurements included total sleep time (TST), time spent in bed (TIB), latency of sleep (LOS), percent sleep efficiency (% SE), sleep architecture (including the time spent in N1, N2, N3 and REM phases expressed as percentage of the total sleep period and the latency of the REM phase). Because in this sleep study we were mainly interested in the REM stage sleep, we only present the sleep data for this stage.

### Resting-state fMRI image acquisition

Resting-state fMRI images and volumetric T1-weighted anatomical images were obtained using a 3T MRI scanner (HDNV Head with a 8-channel head coil, GE Healthcare, Signa HDxt, USA). The gradient strength was 50 mT/m and slew-rate was 150 T/m/s. All scans were acquired between approximately 9 p.m. and 10 p.m. each time. Functional whole-brain images were acquired using a single-shot T2* gradient-echo Echo Planar Imaging (EPI) sequence, interleaved (from bottom to up) acquisition, TR/TE = 2000/30ms, slices = 30, flip angle = 90°, thickness/slice gap = 5/0mm, matrix = 64 × 64, voxel size = 3.75 × 3.75 × 5.0 mm^3^. We acquired 240 functional volumes per run. Each subject underwent only one run each time. Each run cost 8 min. The T1-weigted anatomical image scanning parameters were acquired using a 3DFSPGR sequence, echo time (TE) = 2.17 ms, repetition time (TR) = 7.18 ms, voxel size: 1.2 × 0.47 × 0.47 mm^3^. All subjects were asked to keep their eyes closed and remain awake and relaxed during the scanning. Foam pads and earplugs were used to minimize head movement and reduce the influence of scanner noise.

### Image preprocessing

Images were processed using the DPARSF toolbox (http://www.restfmri.net/forum/DPARSF) [64]. This toolbox was based on Statistical Parametric Mapping 8 (SPM8) (http://www.fil.ion.ucl.ac.uk/spm) and implemented in Matlab (MathWorks Inc., Natick, MA, USA). We performed the following preprocessing steps: (1) the first 10 volumes images were removed for signal equilibrium and adaptation of the participant to the scanning environment; (2) slice-timing correction was performed for adjusting differences in slice acquisition times and the slice at the mid-point of each TR as reference slice; (3) head movement correction was achieved by realignment, the first scan from each volume was aligned to the first scan of the first volume. Then the images within each volume were aligned to the first image of the volume. Any of X, Y and Z direction translation was less than 2 mm; and any of roll, pitch and raw direction rotation was less than 2 degrees. The participant was excluded if any part of volume in this subject was beyond this criterion; (4) spatial normalization to the Montreal Neurological Institute (MNI) stereotactic standard space EPI template through 12-parameter affine transformation (resampling voxel size = 3mm × 3mm × 3mm through trilinear interpolation); (5) images were spatially smoothed using an isotropic Gaussian kernel of 6 mm full width at half-maximum (FWHM); (6) the time courses of various covariates were extracted as regressors of no interest to remove the potential influence of physiological artefacts (including white matter, cerebrospinal fluid, global signal and six motion parameters for head movement; here white matter and cerebrospinal fluid time course extraction’ masks were based upon the a-priori maps available in SPM8). The 24-parameter model containing 6 head motion parameters from the previous image volume, 6 current head motion parameters and 12 corresponding squared items was utilized to reduce effects to BLOD signal induced by head micro movements [65].

### ALFF analysis

ALFF as processed using the DPARSF toolbox pipeline procedure (http://www.restfmri.net/forum/DPARSF). The pre-processed data were subjected to detrending and band pass filtering (0.01–0.08Hz). Following previously reported procedures [66], the filtered time series were transformed to the frequency domain by fast Fourier transform (taper percentage = 0, fast Fourier transform length = shortest). Then, the power spectrum for each voxel was estimated. The power spectrum is proportional to the square of its amplitude in the original time series; therefore, AFLL was calculated by averaging the square root of the power spectrum within 0.01–0.08 Hz in each voxel. For standardization purposes, the ALFF of each voxel was divided by the global mean ALFF values within the priori grey matter mask to standardize the data across different subjects.

### Statistical analysis

Statistical analyses of ALFF were performed in SPM8. A two-sample *t*-test was conducted in SPM8 to determine whether there were any significant regional ALFF differences between the CPI and NS groups. To control for possible confounding effects, variables such as age, gender, education, grey matter volume, mean head translation motion, mean head rotation motion and the SAS scores were entered as covariates in the GLM *t*-test model. The significance threshold was set at *p* < 0.05 (a combination threshold of voxel level at *p* < 0.01 and a cluster size > 147 voxels, which corresponded to a corrected *p* < 0.05 using AlphaSim correction). AlphaSim correction was conducted using the REST toolkit [67].

In order to further explore whether these alterative ALFF regions had any correlation with PSG index, we only did association analysis in patient group. Those clusters mean ALFF ( which values differed significantly between the CPI and NS groups) were extracted using the REX toolbox (http://web.mit.edu/swg/software.htm). Before correlation analysis we checked those ALFF value extracted from previous step and PSG index in order to make sure all data or variables value should less than 3 standard score(Z transformation). If any variable value was more 3 standard deviation it would be moved. Then, the Pearson correlation was used to compute the correlation between these ALFF alterative regions and sub-items of PSG using IBM SPSS Statistics 22 software (http://www-01.ibm.com/software/analytics/spss/).

## Abbreviations

CPI: chronic primary insomnia
ALFF: the amplitude of low-frequency fluctuations
REM sleep: rapid eye movement
NREM: non rapid eye movement
PSG: polysomnography
ARAS: the ascending reticular activating system
SAS: Self-Rating Anxiety Scale
PSQI: Pittsburgh Sleep Quality Index
TST: total sleep time
TIB: time spent in bed
LOS: latency of sleep
% SE: percent sleep efficiency
